# Leukemia stem cells in acute myeloid leukemia: biology, therapeutic resistance, and barriers to durable remission

**DOI:** 10.46439/biomedres.7.083

**Published:** 2026

**Authors:** Giovannino Silvestri

**Affiliations:** 1 Marlene and Stewart Greenebaum Comprehensive Cancer Center, Baltimore, MD 21201, USA; 2 Member, Cancer Therapeutics Program, Marlene and Stewart Greenebaum Comprehensive Cancer Center, Baltimore, MD 21201, USA; 3 Department of Medicine, University of Maryland School of Medicine, Baltimore, MD 21201, USA

## Introduction

Acute myeloid leukemia (AML) is a biologically heterogeneous hematologic malignancy characterized by uncontrolled proliferation of immature myeloid cells and disruption of normal hematopoiesis. Despite major advances in genomic classification and the development of targeted therapies, long-term survival remains limited for many patients due to relapse and treatment resistance [1–3]. Despite therapeutic advances, 5-year overall survival remains approximately 30% in adult AML, underscoring the need for strategies that effectively target disease persistence. Increasing evidence indicates that relapse is driven by a rare subpopulation of leukemia-initiating cells known as leukemic stem cells (LSCs), which possess self-renewal capacity and the ability to reconstitute leukemia following therapy [4–7].

The concept of leukemic stem cells was first demonstrated in seminal xenotransplantation experiments showing that only a small subset of AML cells could initiate leukemia when transplanted into immunodeficient mice [6,7]. These cells display several features reminiscent of normal hematopoietic stem cells, including quiescence, self-renewal, and hierarchical differentiation. Subsequent studies have confirmed that AML is organized as a cellular hierarchy in which LSCs reside at the apex and generate downstream leukemic progenitors and blasts [8–10].

The persistence of LSCs during therapy is considered one of the principal drivers of disease relapse. Unlike bulk leukemic cells, LSCs possess intrinsic mechanisms that confer resistance to chemotherapy, including enhanced DNA repair, altered metabolism, and protective interactions with the bone marrow microenvironment [11–14]. Understanding these mechanisms has therefore become central to the development of curative therapies for AML.

**Figure 1.**
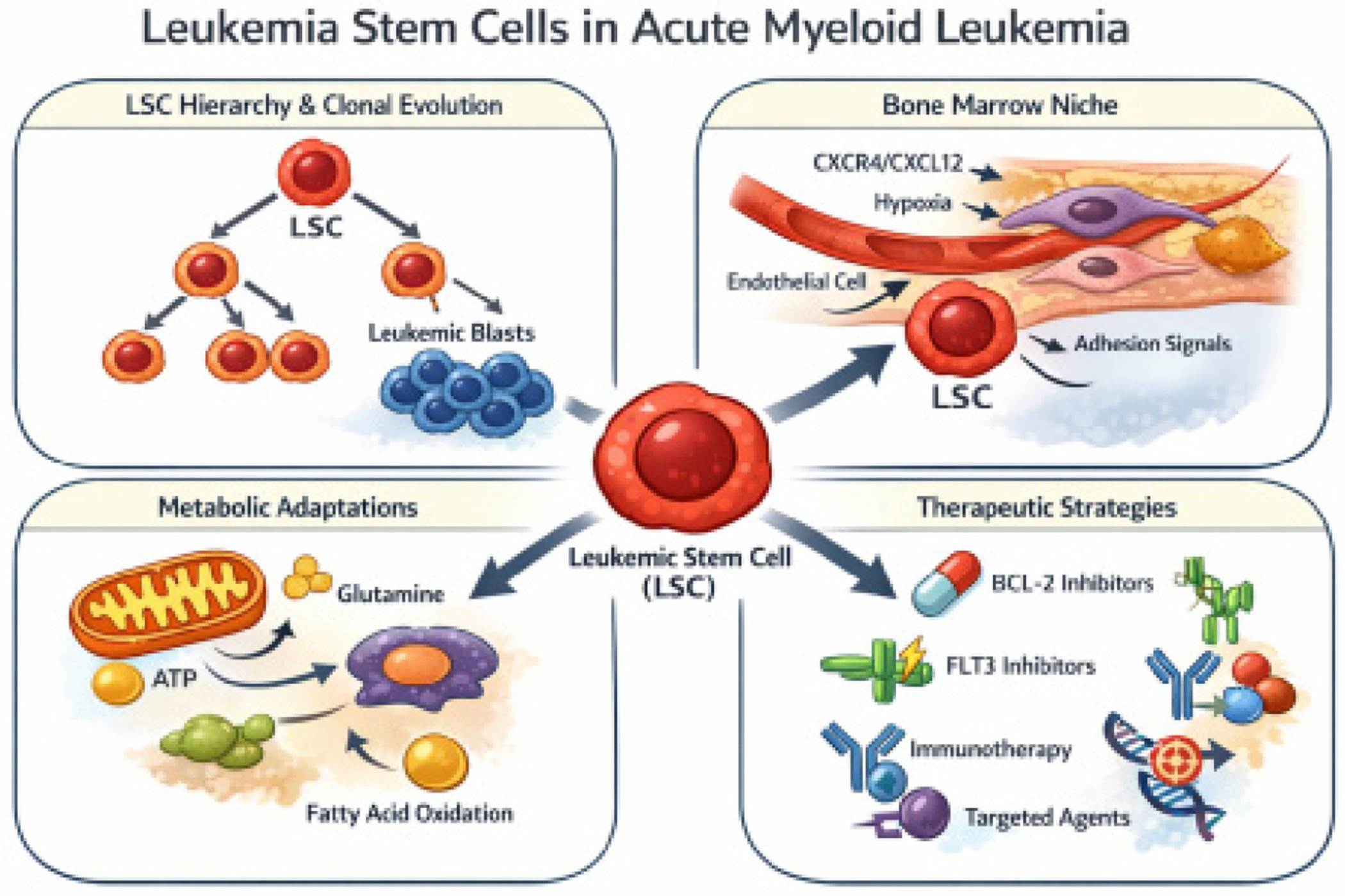
Conceptual model of leukemic stem cell biology and therapeutic resistance in AML.

A conceptual overview of leukemic stem cell biology, including hierarchical organization, metabolic adaptations, microenvironmental interactions, and therapeutic resistance, is illustrated in Figure 1.

AML is hierarchically organized, with leukemic stem cells (LSCs) at the apex generating progenitor populations and bulk leukemic blasts. LSCs reside within the bone marrow microenvironment, where interactions with stromal and endothelial components, along with adhesion signals and chemokine axes, promote retention and survival. Within this niche, hypoxic conditions and microenvironmental cues drive metabolic reprogramming, characterized by increased reliance on mitochondrial oxidative phosphorylation (OXPHOS), glutamine metabolism, and lipid utilization. These adaptations support LSC quiescence and enhance resistance to cytotoxic and targeted therapies. LSCs further evade therapy through intrinsic mechanisms, including altered apoptotic priming and activation of pro-survival signaling pathways. As a result, conventional and targeted treatments often eliminate bulk leukemic cells but fail to eradicate the LSC compartment, leading to disease persistence and relapse. Therapeutic strategies aimed at durable remission must therefore integrate approaches targeting metabolic dependencies, niche interactions, and survival pathways within LSCs.

### Origin and Hierarchical Organization of Leukemic Stem Cells

AML arises through a multistep process involving the accumulation of genetic and epigenetic alterations in hematopoietic stem or progenitor cells. Early mutations may occur in long-lived hematopoietic stem cells, creating pre-leukemic clones that persist over time and provide a reservoir for further leukemogenic events [15–18]. Additional mutations affecting signaling pathways, transcription factors, and epigenetic regulators ultimately give rise to fully transformed leukemic stem cells capable of sustaining malignant hematopoiesis.

Functional studies have demonstrated that LSCs maintain the hierarchical organization of AML. These cells generate more differentiated leukemic progenitors, which in turn produce the bulk population of leukemic blasts observed in patients [19–22]. Importantly, only the LSC population retains long-term self-renewal capacity, making it the critical cellular target for effective therapy.

Clonal evolution further contributes to the complexity of AML stem cell biology. Distinct LSC subclones may coexist within a single patient, each characterized by unique mutational profiles and therapeutic sensitivities [23]. This heterogeneity contributes to treatment resistance and underscores the need for therapeutic strategies capable of targeting multiple LSC populations simultaneously.

Recent single-cell sequencing approaches have further highlighted the functional and phenotypic heterogeneity of LSC populations, including both CD34^+^ and CD34^−^ subsets, with distinct transcriptional and therapeutic profiles.

### Metabolic Adaptations of Leukemic Stem Cells (Table 1)

A defining feature of leukemic stem cells is their unique metabolic profile. While rapidly proliferating leukemic blasts often rely on glycolysis, LSCs depend predominantly on mitochondrial oxidative phosphorylation for energy production [24–26]. This metabolic state has been associated with increased sensitivity to therapies targeting apoptotic priming, including BCL-2 inhibition. This metabolic program supports the quiescent state of LSCs and enhances their resistance to conventional chemotherapy.

Glutamine metabolism plays a particularly important role in maintaining mitochondrial function and redox homeostasis in AML cells. Recent studies have demonstrated that targeting glutamine metabolism can selectively impair leukemic stem cell survival while sparing normal hematopoietic stem cells [27–29]. In addition, alterations in lipid metabolism and fatty acid oxidation contribute to metabolic flexibility and allow LSCs to adapt to changing microenvironmental conditions within the bone marrow niche [30–32].

Importantly, these metabolic dependencies distinguish LSCs from normal hematopoietic stem cells and provide a therapeutic window for selective targeting.

These metabolic adaptations are closely interconnected with signals derived from the bone marrow microenvironment, highlighting the dynamic interplay between intrinsic cellular programs and extrinsic niche factors in sustaining leukemic stem cell survival.

### Leukemic Stem Cells and the Bone Marrow Microenvironment (Table 2)

The bone marrow microenvironment provides a specialized niche that supports leukemic stem cell survival and protects them from therapeutic stress. Interactions between LSCs and stromal cells, endothelial cells, and extracellular matrix components create a protective environment that promotes leukemia progression [33–36].

Additional mechanisms, including CXCR4–CXCL12 signaling, adhesion pathways such as VLA-4/VCAM-1, and stromal-derived soluble factors, further reinforce LSC retention and survival within the niche.

These niche interactions regulate key signaling pathways involved in stem cell maintenance, including Hedgehog, Wnt, and Notch signaling pathways. Activation of these pathways contributes to LSC self-renewal and therapeutic resistance [37–38]. Moreover, hypoxic conditions within the bone marrow niche further promote leukemic stem cell survival by inducing metabolic adaptations and altering transcriptional programs [39–40].

These interactions not only support LSC maintenance but also contribute to metabolic reprogramming and resistance to therapy.

### Immunophenotypic Identification of Leukemic Stem Cells (Table 3)

Leukemic stem cells are commonly enriched within the CD34^+^CD38^−^ compartment, although significant phenotypic heterogeneity exists among patients. Additional markers such as CD123, TIM-3, and CLL-1 have been identified that help distinguish LSCs from normal hematopoietic stem cells [11–13].

Advances in multiparametric flow cytometry and molecular diagnostics have enabled more precise detection and quantification of LSC populations in patient samples. These approaches are increasingly used for monitoring measurable residual disease and predicting relapse risk [37–40].

These markers are increasingly being explored as therapeutic targets and for measurable residual disease monitoring, with potential implications for clinical decision-making.

### Therapeutic Strategies Targeting Leukemic Stem Cells

Targeting leukemic stem cells is considered essential for achieving durable remission in AML. Several therapeutic approaches are currently under investigation, including metabolic inhibitors, epigenetic therapies, and immunotherapeutic strategies [16–18].

Recent advances include therapies targeting apoptotic pathways (e.g., BCL-2 inhibitors such as venetoclax), kinase-driven signaling (e.g., FLT3 inhibitors), metabolic vulnerabilities, and immunotherapeutic approaches targeting LSC-associated antigens.

Metabolic targeting has emerged as a promising strategy due to the dependence of LSCs on oxidative phosphorylation. Agents that disrupt mitochondrial metabolism have demonstrated the ability to selectively impair LSC survival in preclinical models [24–26].

In addition, immunotherapies targeting LSC-associated antigens are being actively explored. Monoclonal antibodies and cellular immunotherapies directed against CD123 and other LSC markers have shown encouraging activity in early clinical studies [37,38].

However, the emergence of resistance and the persistence of functionally diverse LSC populations remain major obstacles to durable disease control.

## Conclusion

Leukemic stem cells represent a central barrier to durable remission in AML due to their capacity for self-renewal, metabolic adaptation, and protection within specialized bone marrow niches.

Emerging therapeutic strategies targeting metabolic dependencies, niche interactions, and survival pathways offer promising avenues for improving outcomes. However, LSC heterogeneity and adaptive resistance remain significant challenges.

Future efforts integrating multi-target approaches and insights from single-cell and multi-omics technologies will be critical to achieving durable disease eradication.

## Figures and Tables

**Table 1.** Major metabolic pathways sustaining AML leukemic stem cells.

Metabolic pathway	Functional role in LSCs	References
Oxidative phosphorylation	Primary energy source for quiescent LSCs	[24–26]
Glutamine metabolism	Supports mitochondrial metabolism and redox balance	[27,28]
Lipid metabolism	Provides metabolic plasticity and survival advantage	[29,30]
Fatty acid oxidation	Adaptation to metabolic stress in the bone marrow niche	[31,32]

**Table 2.** Major components of the AML leukemic stem cell niche.

Niche component	Function in AML stem cell biology	References
Mesenchymal stromal cells	Provide survival and anti-apoptotic signals	[33,34]
Endothelial cells	Maintain vascular niche supporting LSCs	[35]
Osteoblastic niche	Regulates stem cell quiescence	[36]
Hypoxic microenvironment	Promotes metabolic adaptation and therapy resistance	[39,40]

**Table 3.** Immunophenotypic markers associated with AML leukemic stem cells.

Marker	Biological significance	References
CD34^+^CD38^−^	Primitive leukemia-initiating phenotype	[11,12]
CD123	IL-3 receptor α chain enriched in AML LSCs	[13]
TIM-3	Immune checkpoint receptor associated with LSC function	[14]
CLL-1	AML-specific antigen under investigation for immunotherapy	[15]
